# Facile Access to Unnatural Dipeptide-Alcohols Based on *cis*-2,5-Disubstituted Pyrrolidines

**DOI:** 10.3390/molecules20022922

**Published:** 2015-02-11

**Authors:** Yan-Yan Jia, Xiao-Ye Li, Ping-An Wang, Ai-Dong Wen

**Affiliations:** 1 Department of Pharmacy, Xijing Hospital, Fourth Military Medical University, Changle West Road 15, Xi’an 710032, China; E-Mail: xjyypharmacy@126.com; 2 Department of Medicinal Chemistry, School of Pharmacy, Fourth Military Medical University, Changle West Road 169, Xi’an 710032, China; E-Mail: lixiaoye@fmmu.edu.cn

**Keywords:** *cis*-2,5-disubstituted pyrrolidine, unnatural dipeptide-alcohol, hydrogenolysis, phenylalaninol

## Abstract

Well-defined unnatural dipeptide-alcohols based on a *cis*-2,5-disubstitued pyrrolidine backbone were synthesized from commercially available starting materials *meso*-diethyl-2,5-dibromoadipate, (*S*)-(−)-1-phenylethylamine, and phenylalaninol. The structures of these unnatural dipeptide-alcohols are supported by HRMS, ^1^H- and ^13^C-NMR spectroscopy. These unnatural dipeptide-alcohols can act as building blocks for peptidomimetics.

## 1. Introduction

The unnatural peptide-alcohols are important building blocks for the construction of peptide derivatives and play a vital role in peptidomimetics [1,2,3,4]. Some natural pepta-antibiotics [5,6] possessing unnatural peptide-alcohol motifs are isolated from fungus, such as leucinostatins [7], culicinins [8] and hirsuitatins [9]. The unnatural peptide-alcohols that possess a pyrrolidine ring are also attractive to chemists and pharmaceutists [10,11]. Pei and colleagues discovered a series of unnatural peptide-alcohols with one *cis*-2,5-disubstituted pyrrolidine backbone as potent dipeptidyl peptidase IV (DPP-IV) inhibitors for potential oral anti-diabetic drugs [12,13]. Colandrea and researchers [14] reported several 2,5-disubstituted pyrrolidine carboxylic acids that are potent, orally active sphingosine-1-phosphate (S1P) receptor agonists. Aurantiamide, the major isolated component from *Zanthoxylum dissitum* and *Aspergillus penicilloides*, contains one phenyalaninol motif and exhibits anti-bacterial, anti-inflammatory, antioxidant, and anti-HIV effects [15]. Furthermore, Yen *et al.* [16], have developed a series of aurantiamide acetate analogs bearing a phenyalaninol group which were used as potent anti-inflammatory agents.

**Figure 1:**
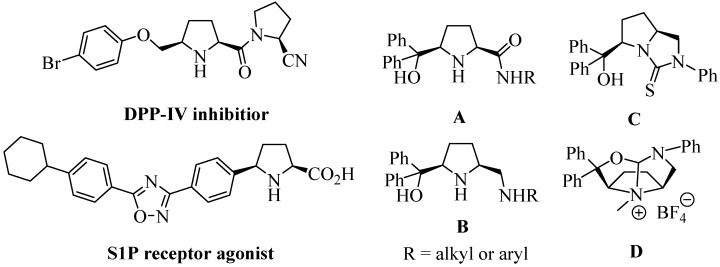
Some novel unsymmetrical *cis*-2,5-disubstituted pyrrolidines.

In our previous work, we reported the efficient construction of enantiopure unsymmetric *cis*-2,5-disubstituted pyrrolidines (Figure 1, compounds **A**–**D**) using *meso*-diethyl-2,5-dibromoadipate and (*S*)-(−)-1-phenylethylamine as starting materials [17,18,19]. Herein, we describe a facile route to unnatural dipeptide-alcohols from phenylalaninol and *cis*-2,5-disubstituted pyrrolidines.

## 2. Results and Discussion

**Scheme 1:**
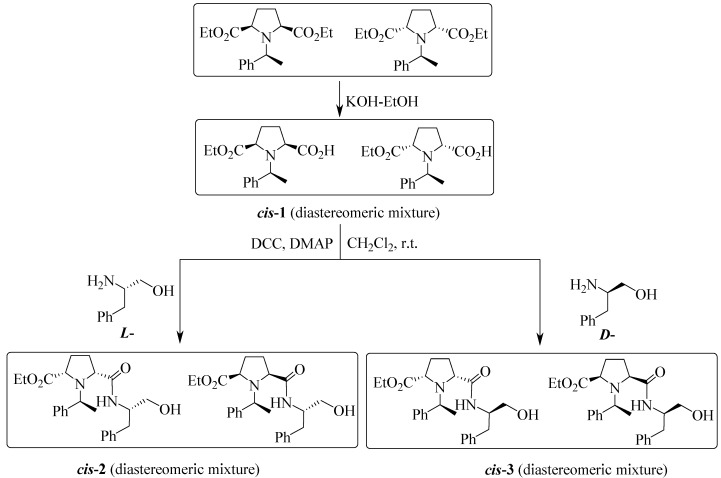
Synthesis of *cis*-**2** and *cis*-**3**.

**Scheme 2:**
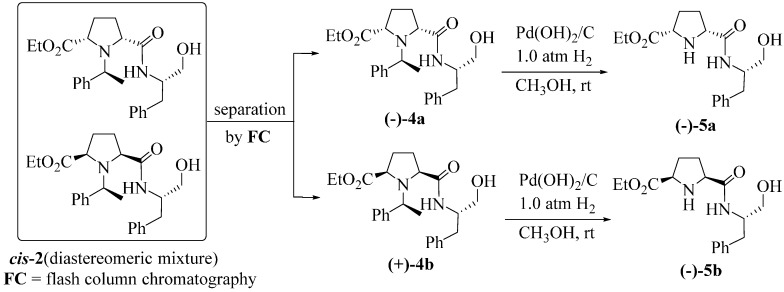
Synthetic routes to **(−)-5a** and **(−)-5b**.

The monoacid *cis*-**1** can be obtained by both chemical and enzymatic protocols via the monohydrolysis of diethyl *cis*-1-[(*S*)-1-phenylethyl]pyrrolidine-2,5-dicarboxylate [20]. In the presence of KOH/EtOH, monoacid *cis*-**1** was obtained in 76% yield as a light yellow slurry. Using the conventional peptide-synthetic protocol, the coupling reactions of l- and d-phenylalaninol with monoacid *cis*-**1** were investigated, respectively. The couplings were performed smoothly by using 1.5 equiv. of dicyclohexylcarbodiimide (DCC) as a coupling reagent in dry CH_2_Cl_2_ at room temperature (rt) (Scheme 1). Both the diastereomers *cis*-**2** and *cis*-**3** were obtained in good yields (up to 80%). Interestingly, the diastereomeric mixture *cis*-**2** prepared from l-phenylalaninol and monoacid *cis*-**1** was easily separated to be **(−)-4a** and **(+)-4b** by a flash column chromatography (FC) on silica gel, however, the diastereomeric mixture *cis*-**3**, the coupling product obtained from monoacid *cis*-**1** and d‑phenylalaninol instead could not be separated as two compounds by flash column chromatography (Scheme 2). The diastereomeric ratio of the major and the minor component in *cis*-**3** is 2/1 which was deduced from its ^1^H-NMR spectrum.

In the presence of catalytic quantity of Pd(OH)_2_/C and under H_2_ atmosphere, compounds **(−)-4a** and **(+)-4b** were converted to be the corresponding deprotected dipeptide-alcohols **(−)-5a** and **(−)-5b** with one protected carboxylic group and one C-terminal hydroxyl group, respectively (Scheme 2). The dipeptide-alcohols **(−)-5a** and **(−)-5b** containing a *cis*-pyrrolidine backbone with one free N-terminal at pyrrolidine ring and one C-terminal hydroxyl group in the side-chain can be used as valuable building blocks for connection of other amino acids to furnish complex peptide-alcohols.

**Scheme 3:**
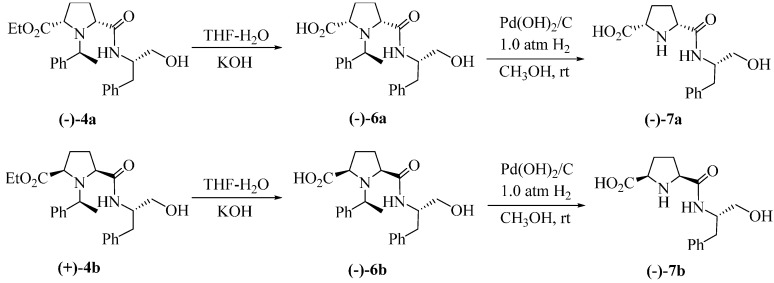
Synthetic routes to **(−)-7a** and **(−)-7b**.

Hydrolysis of compounds **(−)-4a** and **(+)-4b** using solid KOH in THF/H_2_O afforded the corresponding dipeptide-alcohols **(−)-6a** and **(−)-6b** with free C-terminal carboxylic acid and hydroxyl groups (Scheme 3). These free carboxylic acid and hydroxyl groups can enable the coupling with other amino acids to yield complex unnatural peptide-alcohols. The other two unnatural dipeptide-alcohols **(−)-7a** and **(−)-7b** with both free C- and N-terminus were obtained by catalytic hydrogenolysis of compounds **(−)-6a** and **(−)-6b** in methanol at room temperature separately.

## 3. Experimental Section

### 3.1. General Information

Melting points are uncorrected and expressed in °C. ^1^H- and ^13^C-NMR spectra were measured in CDCl_3_, MeOD or DMSO-*d*_6_ solution on a Bruker AV-300 or AV-500 spectrometers (Bruker, Fällanden, Switzerland) using TMS as an internal reference. Multiplicities are designated by the following abbreviations: s, singlet; d, doublet; t, triplet; q, quartet; br, broad; m, multiplet. Optical rotations analyses were performed on a Model 343 Polarimeter (Perkin-Elmer, Waltham, MA, USA). High-resolution mass spectra were performed on a VG Micromass 7070F Mass Spectrometer (VG Instruments, St Leonards-on-Sea, UK) with ES ionization (ESI). All commercially available reagents were used as received. Products were purified by flash column chromatography on silica gel purchased from Qingdao Haiyang Chemical Co. Ltd. (Qingdao, China). All reactions involving air or moisture sensitive species were performed in oven-dried glassware under inert atmosphere. The monoacid *cis*-**1** was prepared following the reported procedures in the previous literature [17].

#### 3.1.1. Typical Procedure for *cis*-**2** or *cis*-**3**

To a mixture of monoacid *cis*-**1** (2.90 g, 10.0 mmol) and *L*-phenylalaninol (1.60 g, 10.5 mmol) in dry CH_2_Cl_2_ (50 mL), DCC (3.20 g, 15.5 mmol) and DMAP (125 mg, 1.0 mmol) added at 0 °C, and the mixture was stirred for 0.5 h at this temperature and stirred overnight at rt. After the reaction was finished, it was filtered on a Celite pad. The solvents was evaporated to give *cis*-**2** (diastereomeric mixture) as a yellow oil which was purified by a flash column chromatography on silica gel to afford **(−)-4a** and **(+)-4b**. The coupling product *cis*-**3** (diastereomeric mixture) was obtained by the similar procedure from d-phenylalaninol (0.79 g, 5.2 mmol) and monoacid *cis*-**1** (1.46 g, 5.0 mmol), and it could not be separated by a flash column chromatography.

*cis-Ethyl 5-{[(R)-1-hydroxy-3-phenylpropan-2-yl]carbamoyl}-1-[(S)-1-phenylethyl]pyrrolidine-2-car-boxylate* (*cis*-**3** diastereomeric mixture). 1.82 g, 85%; light yellow wax. ^1^H-NMR (500 MHz, CDCl_3_): Major isomer: δ_H_ 1.25 (t, *J* = 7.0 Hz, 2H), 1.34 (d, *J* = 5.5 Hz, 2H), 1.79–2.11 (m, 2.5 H), 2.82–3.02 (m, 2H), 3.45–3.48 (m, 1H), 3.55–3.68 (m, 3H), 3.81–3.87 (m, 1H), 3.99–4.08 (m, 1H), 4.11–4.16 (m, 1H), 7.07–7.34 (m, 10H), 8.46 (d, *J* = 8.5 Hz, 0.66H). Minor isomer: δ 1.18 (t, *J* = 7.5 Hz, 1H), 8.88 (d, *J* = 9.0 Hz, 0.33H), the other signals are overlapped with the major isomer. HRMS (ESI) *m/z* calcd for C_25_H_33_N_2_O_4_ (MH^+^): 425.2440. Found: 425.2451.

*(2S,5R)-Ethyl 5-{[(S)-1-hydroxy-3-phenylpropan-2-yl]carbamoyl}-1-[(S)-1-phenylethyl]pyrrolidine-2-carboxylate* **(−)-4a**. 1.53 g, 37%; light yellow solid, mp 89.5–91 °C, R*_f_* = 0.50 (*n*-hexane/EtOAc, 2:1), ${\left[\alpha \right]}_{\text{ D}}^{20}$ −71.6 (*c* 0.5, CHCl_3_). ^1^H-NMR (300 MHz, CDCl_3_): δ_H_ 1.28 (t, *J* = 7.2 Hz, 3H), 1.38 (d, *J* = 6.9 Hz, 3H), 1.79–1.87 (m, 2H), 1.91–2.01 (m, 2H), 2.75 (br, 1H), 2.89 (dd, *J*_1_ = 8.4 Hz, *J*_2_ = 5.7 Hz, 1H), 3.02 (dd, *J*_1_ = 7.5 Hz, *J*_2_ = 6.6 Hz, 1H), 3.61–3.76 (m, 4H), 3.86 (q, *J* = 6.9 Hz, 1H), 4.16 (q, *J* = 7.2 Hz, 2H), 4.20–4.30 (m, 1H), 7.17–7.33 (m, 10H), 8.65 (d, *J* = 8.1 Hz, 1H). ^13^C NMR (300 MHz, CDCl3): δ_C_ 14.3, 19.4, 30.2, 30.7, 36.8, 52.8, 61.1, 61.5, 64.0, 65.1, 65.4, 126.4, 127.6, 127.7, 128.3, 128.5, 129.2, 138.2, 142.3, 175.9, 176.3. HRMS (ESI) *m/z* calcd for C_25_H_33_N_2_O_4_ (MH^+^): 425.2440. Found: 425.2458.

*(2R,5S)-Ethyl 5-{[(S)-1-hydroxy-3-phenylpropan-2-yl]carbamoyl}-1-[(S)-1-phenylethyl]pyrrolidine-2-carboxylate* **(+)-4b**. 1.91 g, 45%; light yellow solid, mp 101.5–103.7 °C, R*_f_* = 0.30 (*n*-hexane/EtOAc, 2:1), ${\left[\alpha \right]}_{\text{ D}}^{20}$ +18.2° (*c* 1.05, CHCl_3_). ^1^H-NMR (300 MHz, CDCl_3_): δ_H_ 1.12 (t, *J* = 7.2 Hz, 3H), 1.26 (d, *J* = 6.9 Hz, 3H), 1.63–1.74 (m, 1H), 1.93–2.01 (m, 3H), 2.87–3.03 (m, 2H), 3.39 (t, *J* = 6.9 Hz, 1H), 3.57–3.78 (m, 5H), 3.88–4.05 (m, 2H), 4.18–4.28 (m, 1H), 7.19–7.35 (m, 10H), 8.81 (d, *J* = 8.1 Hz, 1H). ^13^C-NMR (300 MHz, MeOD) δ_C_ 13.1, 20.1, 30.1, 30.7, 36.6, 52.3, 60.7, 62.8, 63.0, 65.2, 66.1, 126.1, 127.2, 128.0, 128.2, 128.9, 138.3, 142.8, 176.7, 176.9. HRMS (ESI) *m/z* calcd for C_25_H_33_N_2_O_4_ (MH^+^): 425.2440. Found: 425.2447.

#### 3.1.2. Typical Procedure for **(−)-5a** or **(−)-5b**

In the presence of Pd(OH)_2_/C (0.20 g), the compound **(−)-4a** (0.50 g, 1.17 mmol) in MeOH (10.0 mL) was stirred overnight under 1.0 atm H_2_ at rt. After the reaction was finished, it was filtered on a Celite pad to remove catalyst. The filtrate was evaporated to give the desired product **(−)-5a** without further purification. Compound **(−)-5b** was obtained from **(+)-4b** by the similar procedure.

*(2S,5R)-Ethyl 5-{[(S)-1-hydroxy-3-phenylpropan-2-yl]carbamoyl}pyrrolidine-2-carboxylate* **(−)-5a**. Light yellow crystals, 0.35 g, 92%, mp 52–53.5 °C, ${\left[\alpha \right]}_{\text{ D}}^{20}$ −210.5° (*c* 1.0, MeOH). ^1^H-NMR (500 MHz, CDCl_3_): δ_H_ 1.31 (t, *J* = 7.0 Hz, 3H), 1.62–1.66 (m, 1H), 1.90–1.94 (m, 1H), 2.11–2.18 (m, 2H), 2.87–2.97 (m, 2H), 3.50–3.65 (m, 1H), 3.71–3.74 (m, 1H), 3.98–4.07 (m, 1H), 4.17–4.30 (m, 3H), 7.19–7.29 (m, 5H), 8.45 (d, *J* = 8.0 Hz, 1H). ^13^C-NMR (300 MHz, CDCl_3_): δ_C_ 13.3, 29.2, 30.7, 36.7, 52.1, 59.9, 60.7, 61.3, 63.3, 125.9, 127.8, 128.9, 138.4, 175.7, 175.9. HRMS (ESI) *m/z* calcd for C_17_H_26_N_2_O_4_ (MH^+^): 321.1814. Found: 321.1832. 

*(2R,5S)-Ethyl 5-{[(S)-1-hydroxy-3-phenylpropan-2-yl]carbamoyl}pyrrolidine-2-carboxylate* **(−)-5b**. Colorless crystals, 0.34 g, 90%, mp 71–73 °C, ${\left[\alpha \right]}_{\text{ D}}^{20}$ −3.6° (*c* 0.5, DMSO). ^1^H-NMR (300 MHz, MeOD): δ_H_ 1.28 (t, *J* = 7.2 Hz, 3H), 1.69–1.87 (m, 2H), 2.0–2.18 (m, 2H), 2.77 (dd, *J* = 8.7, 5.1 Hz, 1H), 2.98 (dd, *J* = 7.8, 5.7 Hz, 1H), 3.56 (t, *J* = 5.7 Hz, 2H), 3.68–3.72 (m, 1H), 3.93 (t, *J* = 7.5 Hz, 1H), 4.05–4.14 (m, 1H), 4.19 (q, *J* = 7.2 Hz, 2H), 7.12–7.23 (m, 5H). ^13^C-NMR (500 MHz, DMSO-*d*_6_): δ_C_ 14.5, 30.1, 31.4, 37.1, 52.4, 60.2, 60.8, 61.4, 62.9, 126.4, 128.6, 129.6, 139.3, 173.8, 175.2. HRMS (ESI) *m/z* calcd for C_17_H_26_N_2_O_4_ (MH^+^): 321.1814. Found: 321.1839.

#### 3.1.3. Typical Procedure for **(−)-6a** or **(−)-6b**

The compound **(−)-4a** (1.0 g, 2.35 mmol) in THF/H_2_O (1:1) (15 mL) was added by KOH pellets (0.33 g, 4.7 mmol) and the mixture was stirred 2 h at rt. After the reaction was finished, the solvent was evaporated and the acidity of the aqueous residue was adjusted to be pH = 2.0 by 6.0 M HCl, then it was extracted by ethyl acetate (3 × 10 mL), the combined organic layer was washed by H_2_O (2 × 5 mL) and brine (10 mL), dried (Na_2_SO_4_). The solvent was evaporated under reduced pressure to give the desired product **(−)-6a** without further purification. Compound **(−)-6b** was obtained from **(−)-4b** by the similar procedure.

*(2S,5R)-5-{[(S)-1-Hydroxy-3-phenylpropan-2-yl]carbamoyl}-1-[(S)-1-phenylethyl]pyrrolidine-2-carboxylic acid* **(−)-6a**. Colorless powder, 0.85 g, 91%, mp 182.5–183.5 °C, ${\left[\alpha \right]}_{\text{ D}}^{20}$ −65.5° (*c* 0.3, MeOH). ^1^H-NMR (300 MHz, MeOD): δ_H_ 1.20 (d, *J* = 9.0 Hz, 3H), 1.24–1.32 (m, 1H), 1.42–1.48 (m, 1H), 1.68–1.92 (m, 2H), 2.59 (dd, *J* = 9.6, 4.5 Hz, 1H), 2.95 (dd, *J* = 9.0, 4.8 Hz, 1H), 3.54 (dd, *J* = 7.2, 1.5 Hz, 1H), 3.81 (q, *J* = 6.6 Hz, 1H), 3.91–4.02 (m, 1H), 4.92 (br, 1H), 7.11–7.29 (m, 10H), 8.42 (d, *J* = 9.0 Hz, 1H). ^13^C-NMR (300 MHz, MeOD): δ_C_ 21.0, 30.0, 30.1, 37.3, 52.2, 61.7, 63.6, 64.6, 65.2, 126.4, 127.6, 128.4, 128.7, 129.4, 139.5, 143.8, 174.3, 177.6. HRMS (ESI) *m/z* calcd for C_23_H_29_N_2_O_4_ (MH^+^): 397.2127. Found: 397.2139.

*(2R,5S)-5-{[(S)-1-Hydroxy-3-phenylpropan-2-yl]carbamoyl}-1-[(S)-1-phenylethyl]pyrrolidine-2-carboxylic acid* **(−)-6b**. Colorless crystals, 0.83 g, 90%, mp 188–190 °C, ${\left[\alpha \right]}_{\text{ D}}^{20}$ −39.2° (*c* 0.25, MeOH). ^1^H-NMR (300 MHz, MeOD): δ_H_ 1.35 (d, *J* = 6.9 Hz, 3H), 1.59–1.72 (m, 1H), 1.83–1.92 (m, 1H), 2.01–2.14 (m, 2H), 2.72 (dd, *J* = 9.6, 4.5 Hz, 1H), 3.04 (dd, *J* = 8.4, 5.7 Hz, 1H), 3.55 (d, *J* = 5.4 Hz, 2H), 3.81 (q, *J* = 7.8 Hz, 2H), 4.06–4.21 (m, 2H), 7.17–7.40 (m, 11H). ^13^C-NMR (300 MHz, MeOD): δ_C_ 20.2, 30.3, 31.2, 37.0, 52.4, 61.7, 63.0, 64.5, 65.9, 126.3, 127.5, 127.8, 128.4, 128.5, 129.3, 129.3, 139.0, 143.0, 175.3, 177.3. HRMS (ESI) *m/z* calcd for C_23_H_29_N_2_O_4_ (MH^+^): 397.2127. Found: 397.2141.

#### 3.1.4. Typical Procedure for the Synthesis of **(−)-7a** or **(−)-7b**

In the presence of Pd(OH)_2_/C (0.23 g), the compound **(−)-6a** (0.60 g, 1.5 mmol) in MeOH (8 mL) was stirred overnight under 1.0 atm H_2_ at rt. After the reaction was finished, it was filtered on a Celite pad to remove catalyst. The filtrate was evaporated to give the desired product **(−)-7a** without further purification. Compound **(−)-7b** was obtained from **(−)-6b** by the similar procedure.

*(2S,5R)-5-{[(S)-1-Hydroxy-3-phenylpropan-2-yl]carbamoyl}pyrrolidine-2-carboxylic acid* **(−)-7a**. Colorless powder, 0.38 g, 87%, mp 197–199 °C, ${\left[\alpha \right]}_{\text{ D}}^{20}$ −36.7° (*c* 0.125, MeOH). ^1^H-NMR (300 MHz, DMSO-*d*_6_): δ_H_ 1.25–1.35 (m, 1H), 1.69–1.77 (m, 1H), 1.91–2.05 (m, 2H), 2.59 (dd, *J* = 9.0, 4.5 Hz, 1H), 2.88 (dd, *J* = 8.7, 5.1 Hz, 1H), 3.55–3.69 (m, 2H), 3.83 (t, *J* = 7.5 Hz, 1H), 3.92–3.99 (m, 1H), 7.14–7.28 (m, 5H), 8.33 (d, *J* = 9.0 Hz, 1H). ^13^C-NMR (500 MHz, DMSO-*d*_6_): δ_C_ 29.8, 30.8, 36.9, 53.2, 60.7, 60.8, 62.9, 126.5, 128.4, 128.6, 129.5, 139.4, 170.9, 173.2. HRMS (ESI) *m/z* calcd for C_15_H_21_N_2_O_4_ (MH^+^): 293.1501. Found: 293.1496.

*(2R,5S)-5-{[(S)-1-Hydroxy-3-phenylpropan-2-yl]carbamoyl}pyrrolidine-2-carboxylic acid* **(−)-7b**. Colorless powder, 0.40 g, 91%, mp 212–214 °C, ${\left[\alpha \right]}_{\text{ D}}^{20}$ −10.2° (*c* 0.2, MeOH). ^1^H-NMR (500 MHz, DMSO-*d*_6_): δ_H_ 1.25–1.35 (m, 1H), 1.70–1.79 (m, 1H), 1.96–2.10 (m, 2H), 2.62 (dd, *J* = 9.0, 4.5 Hz, 1H), 2.89 (dd, *J* = 8.5, 5.0 Hz, 1H), 3.10–3.60 (m, 1H, overlap), 3.88 (t, *J* = 7.0 Hz, 1H), 3.90–4.02 (m, 1H), 4.93 (br, 1H), 7.16–7.27 (m, 5H), 8.42 (d, *J* = 8.0 Hz, 1H). ^13^C-NMR (500 MHz, DMSO-*d*_6_): δ_C_ 29.9, 30.6, 37.1, 53.2, 60.6, 61.0, 63.2, 126.5, 128.5, 129.6, 139.2, 169.8, 172.2. HRMS (ESI) *m/z* calcd for C_15_H_21_N_2_O_4_ (MH^+^): 293.1501. Found: 293.1510.

## 4. Conclusions

A facile route to unnatural dipeptide-alcohols based on a *cis*-2,5-disubstituted pyrrolidine backbone that is readily prepared from commercially available materials is described. Two distereomers are separated by simple flash column chromatography, and these unnatural peptide-alcohols contain a free C-terminus, a C-terminal hydroxyl group or a N-terminus that can facilitate couplings with other amino acids to give more complex polypeptide alcohols.

## Supplementary Materials

Supplementary materials can be accessed at: http://www.mdpi.com/1420-3049/20/02/2922/s1.
