# Supplementary material for: Facile Access to Unnatural Dipeptide-Alcohols Based on cis-2,5-Disubstituted Pyrrolidines
Source: Molecules. 2015 Feb 11;20(2):2922–30. doi: 10.3390/molecules20022922 (PMC6272547; doi:10.3390/molecules20022922)
Supplement: Supplementary file 1 [file molecules-20-02922-s001.pdf]

# Supplementary


| | |
|---|---|
| $^{1}$H-NMR of ***cis*-1** and ***cis*-3** | **S1** |
| $^{1}$H- and $^{13}$C-NMR of **(−)-4a** | **S2** |
| $^{1}$H- and $^{13}$C-NMR of **(+)-4b** | **S3** |
| $^{1}$H- and $^{13}$C-NMR of **(−)-5a** | **S4** |
| $^{1}$H- and $^{13}$C-NMR of **(−)-5b** | **S5** |
| $^{1}$H- and $^{13}$C-NMR of **(−)-6a** | **S6** |
| $^{1}$H- and $^{13}$C-NMR of **(−)-6b** | **S7** |
| $^{1}$H- and $^{13}$C-NMR of **(−)-7a** | **S8** |
| $^{1}$H- and $^{13}$C-NMR of **(−)-7b** | **S9** |

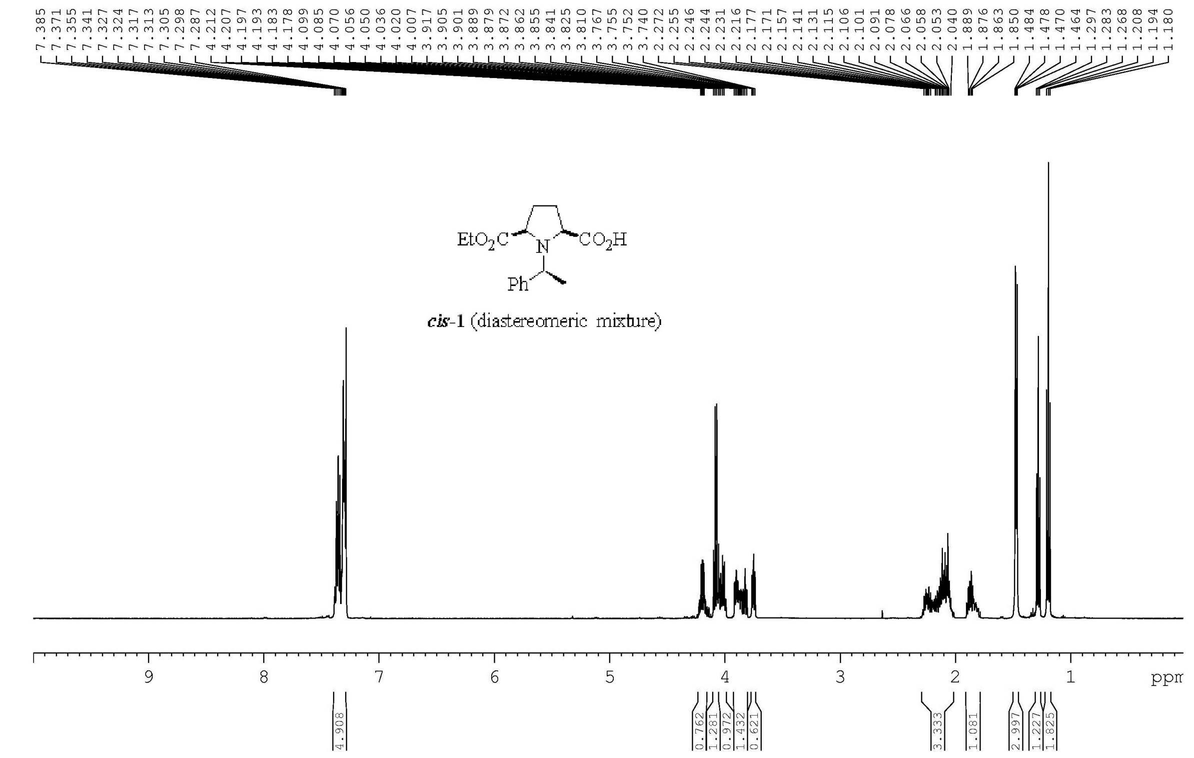


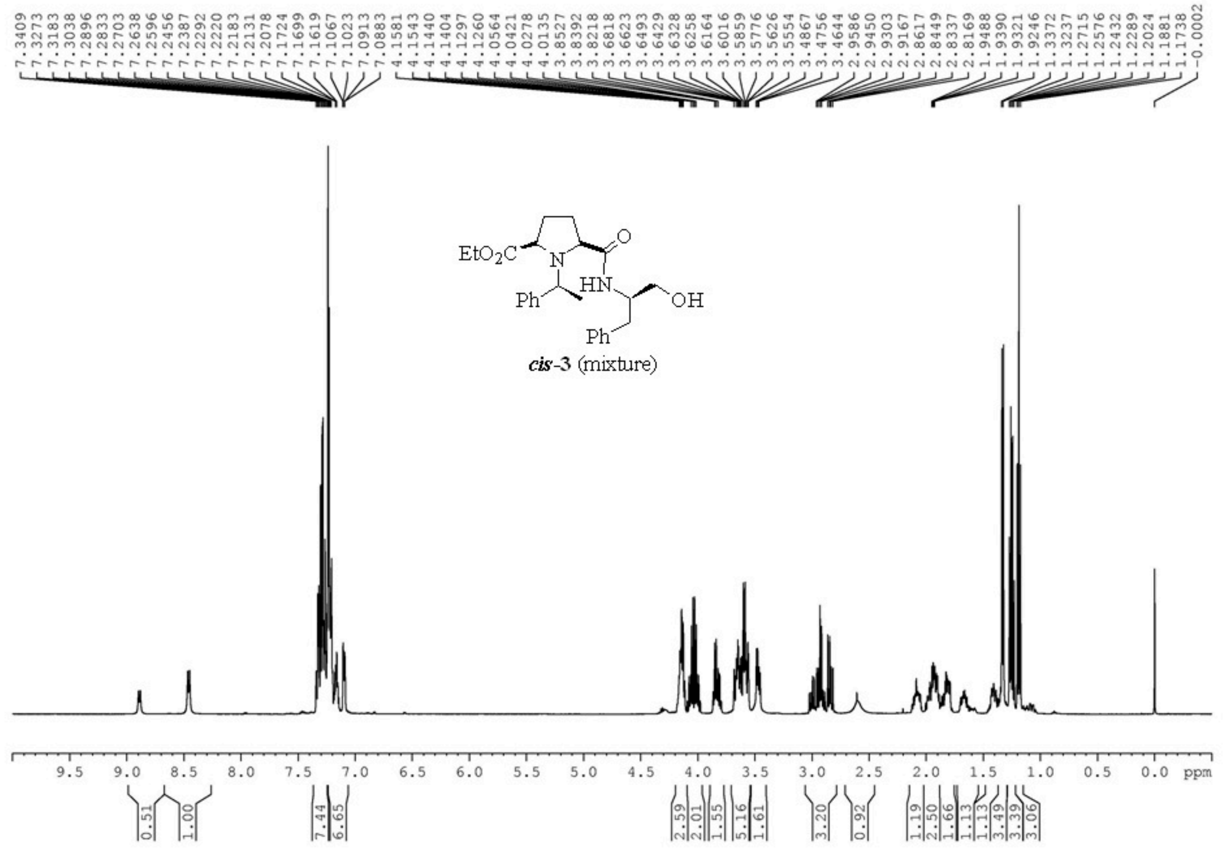


**Figure S1.** $^{1}$H NMR of ***cis*-1** and ***cis*-3**.

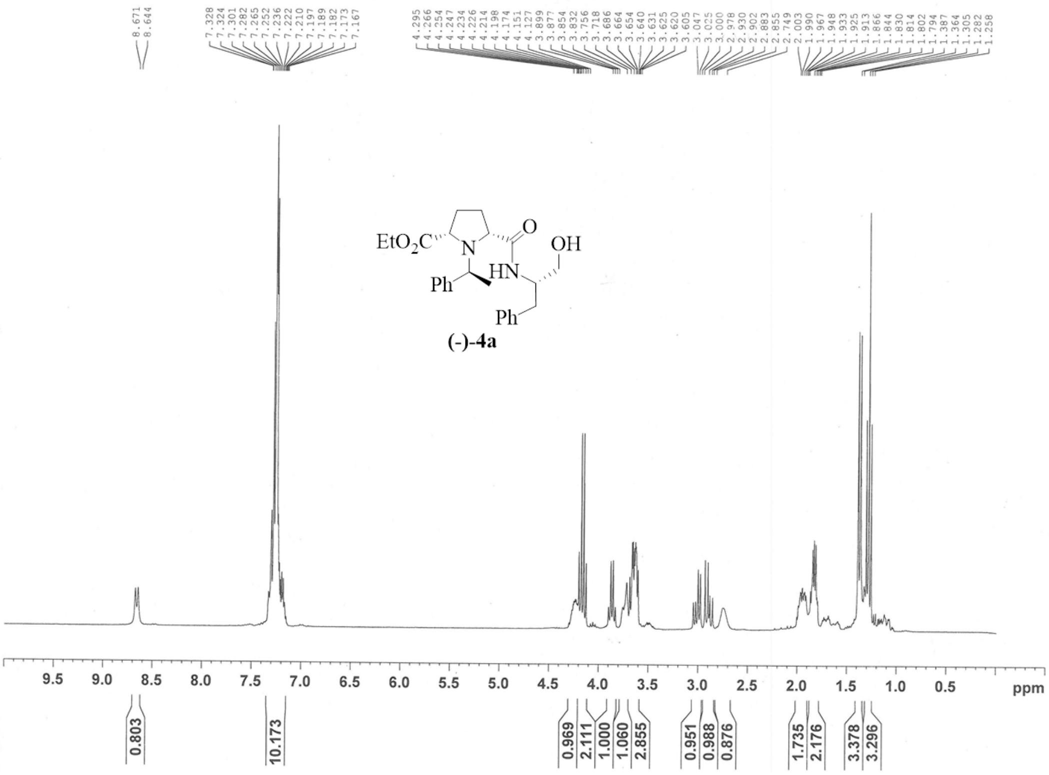


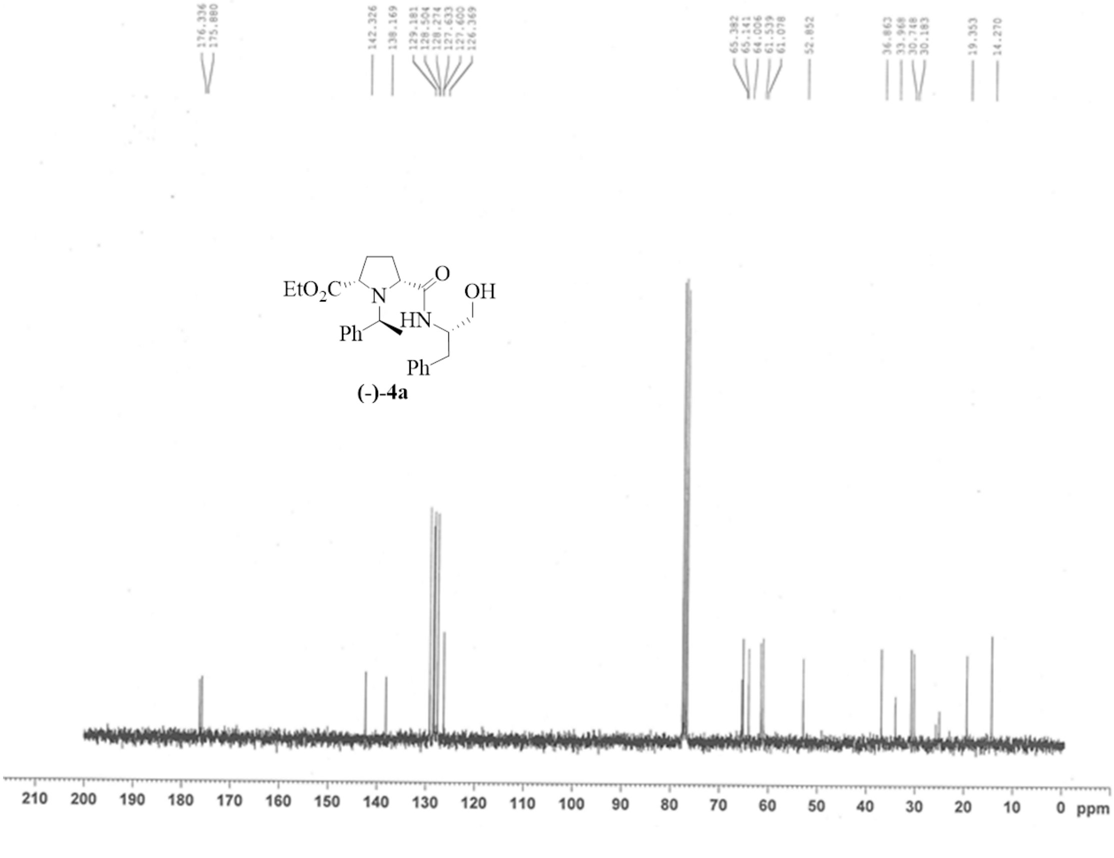


**Figure S2.** $^{1}$H and $^{13}$C NMR of **(−)-4a**.

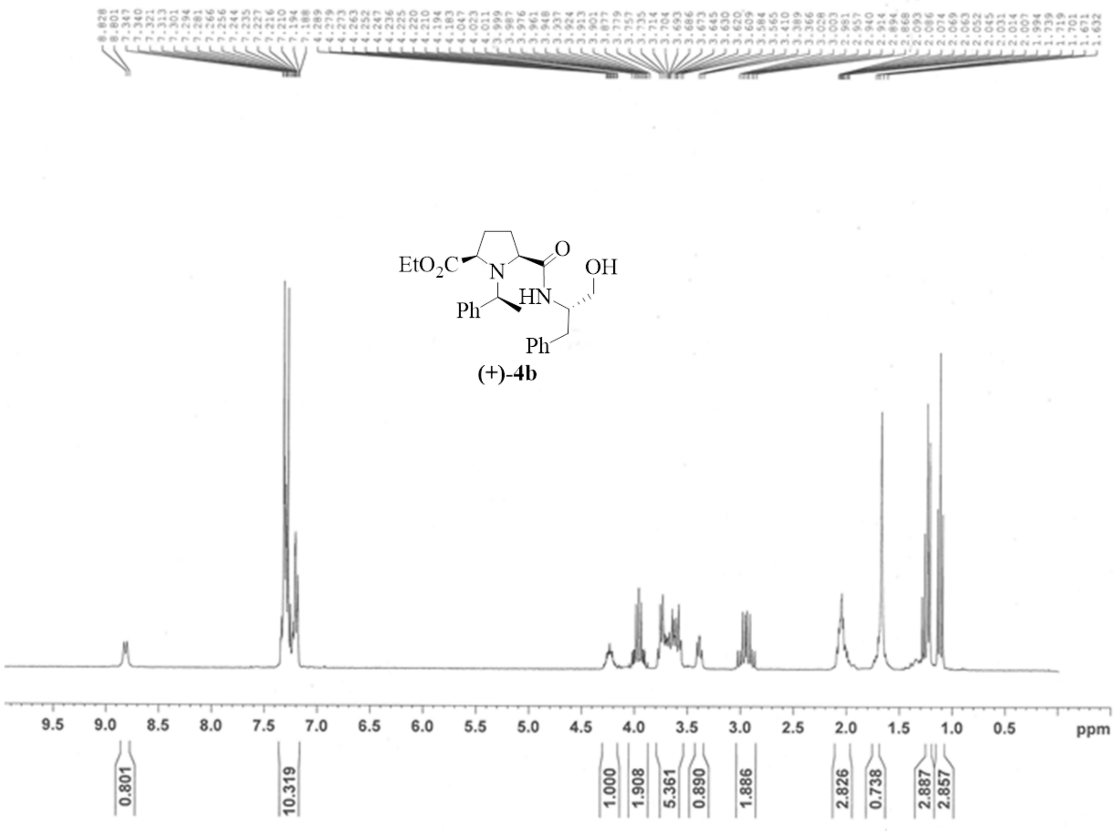


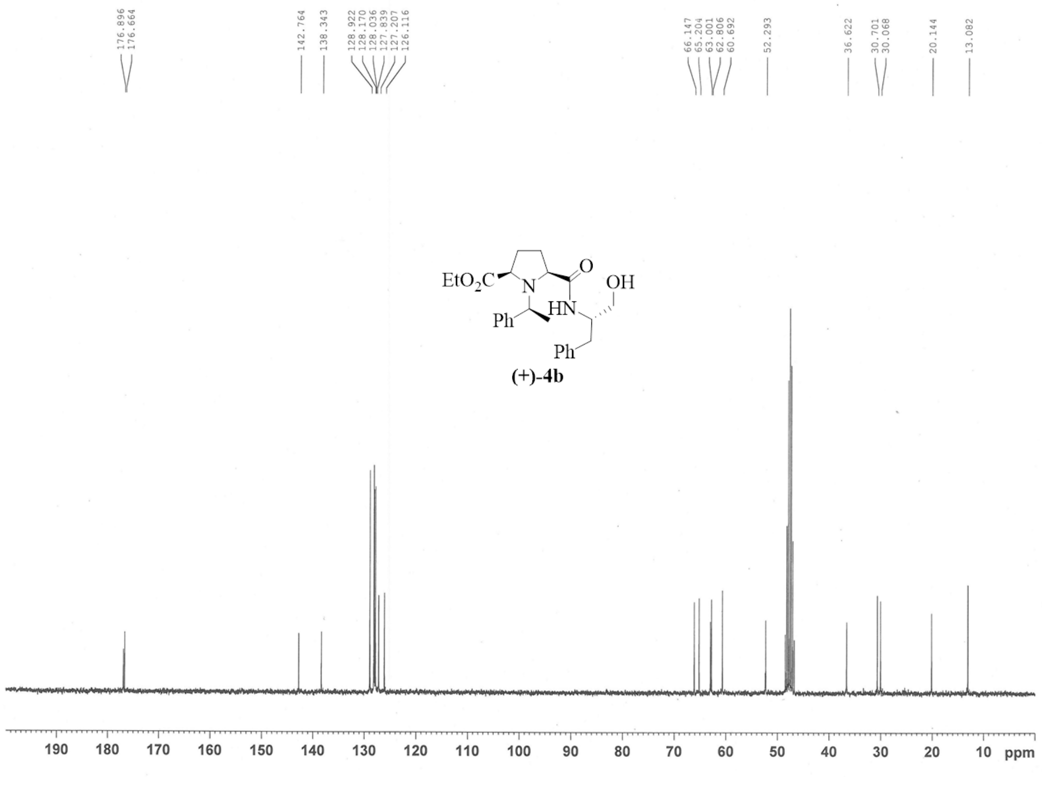


**Figure S3.** $^{1}H$ and $^{13}C$ NMR of **(+)-4b**.

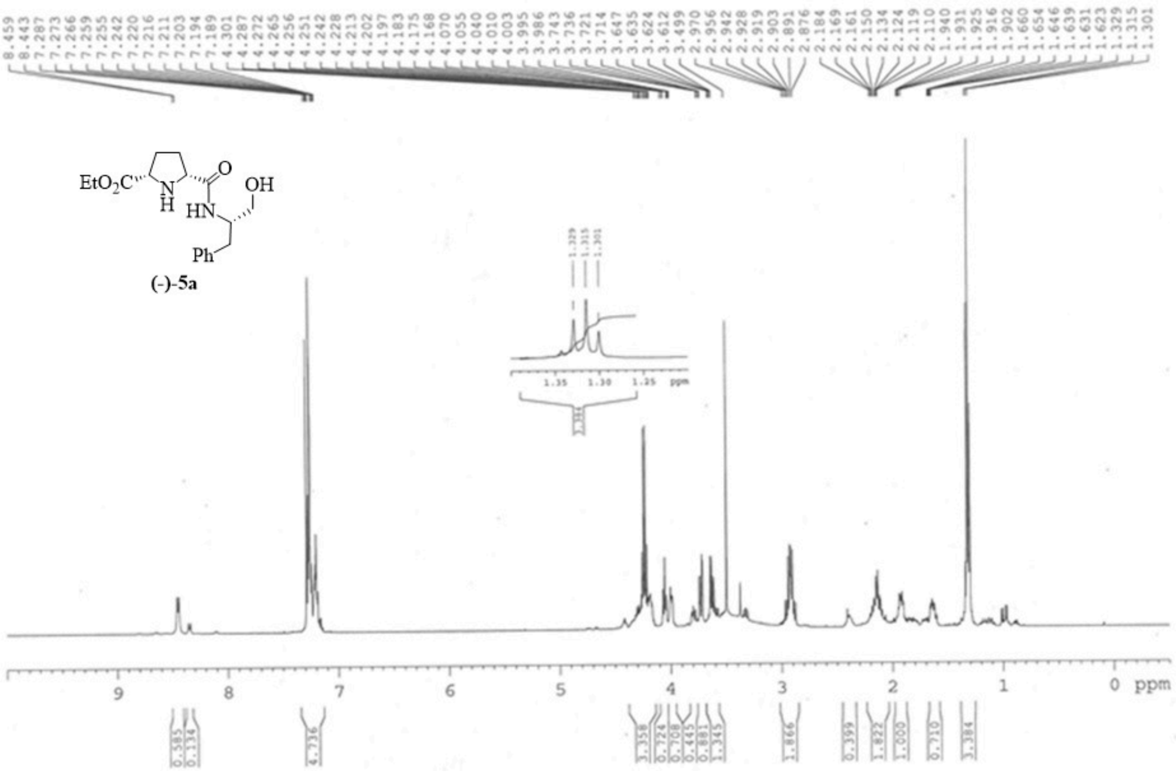

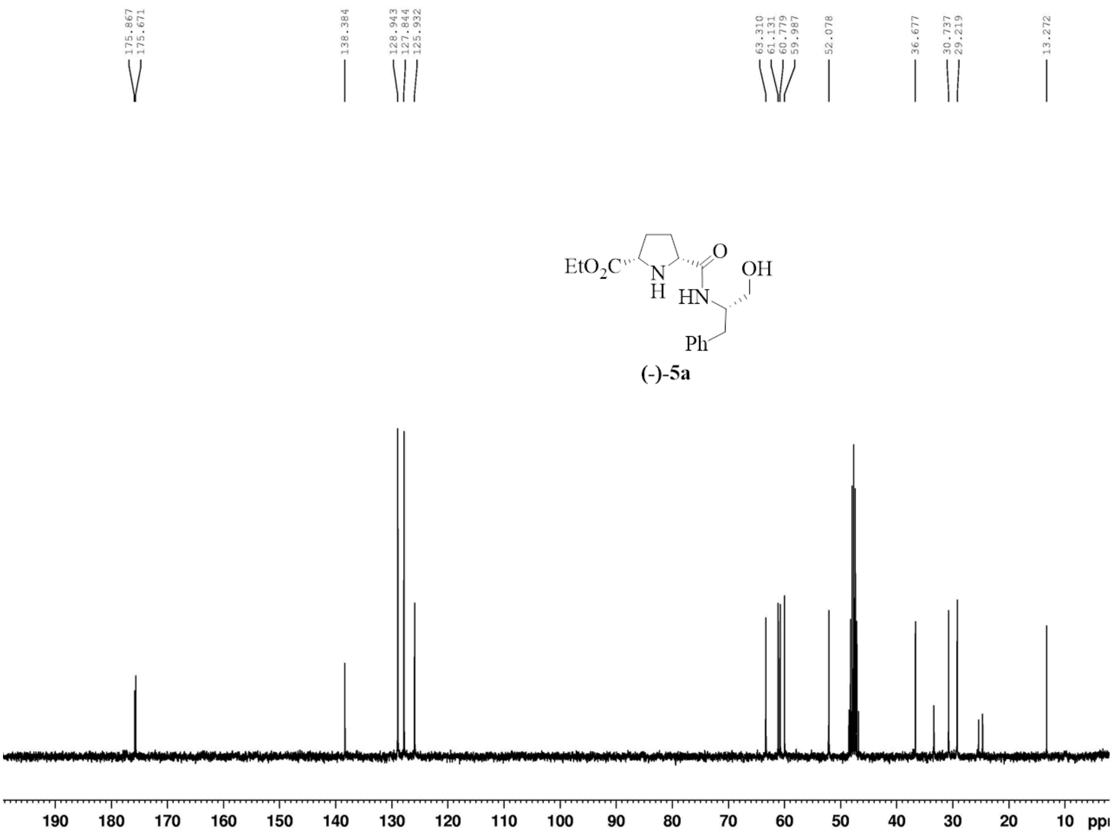

**Figure S4.** $^{1}$H and $^{13}$C NMR of **(−)-5a**.

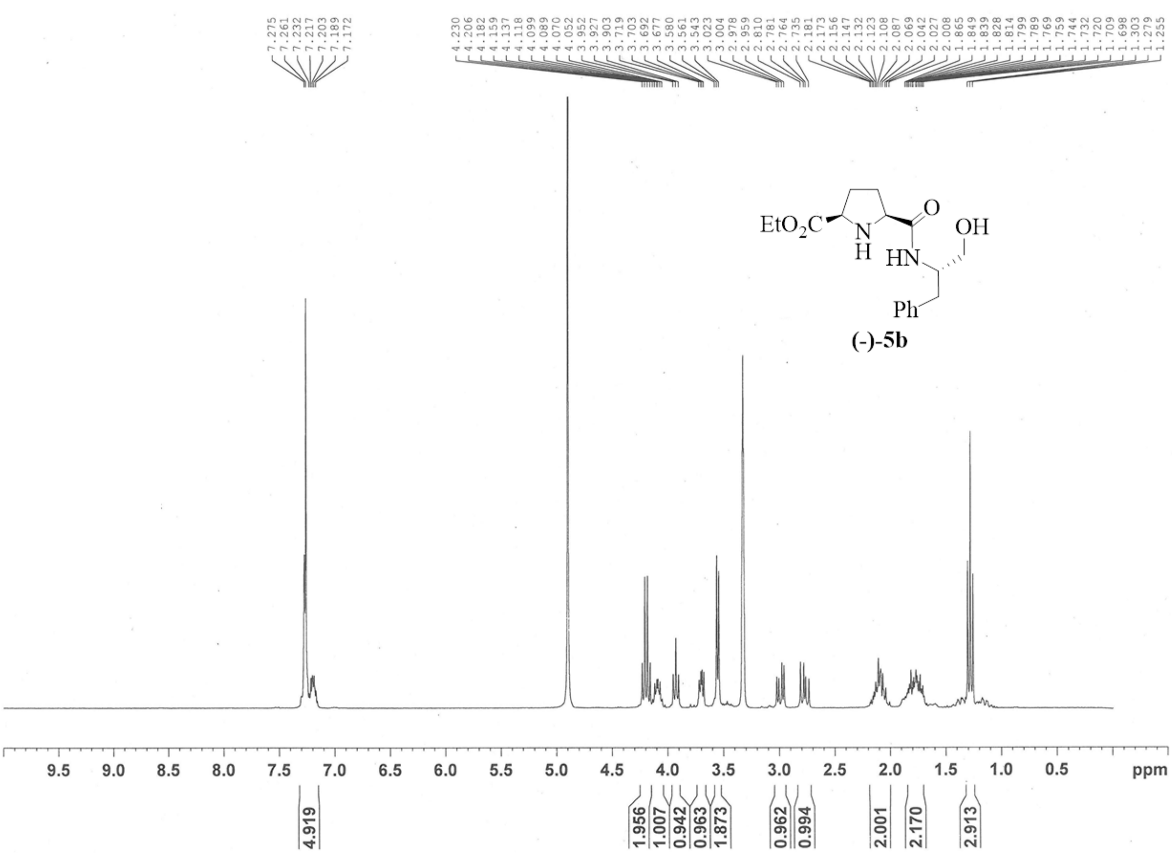


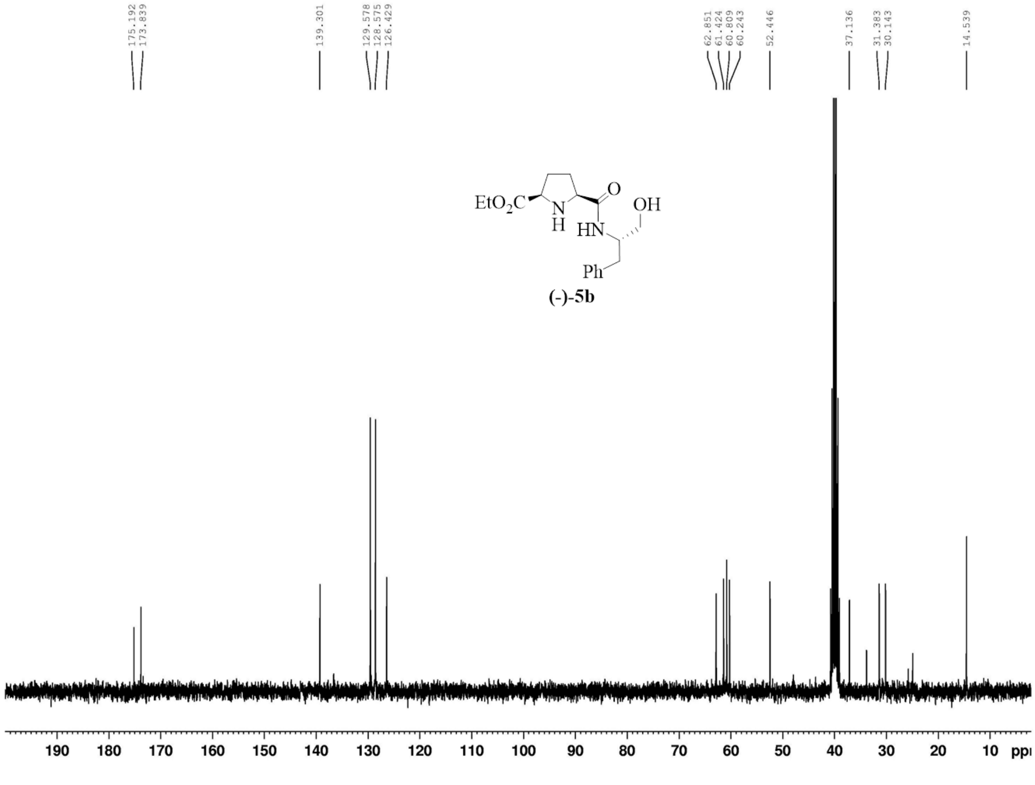


**Figure S5.** $^{1}H$ and $^{13}C$ NMR of **(−)-5b**.

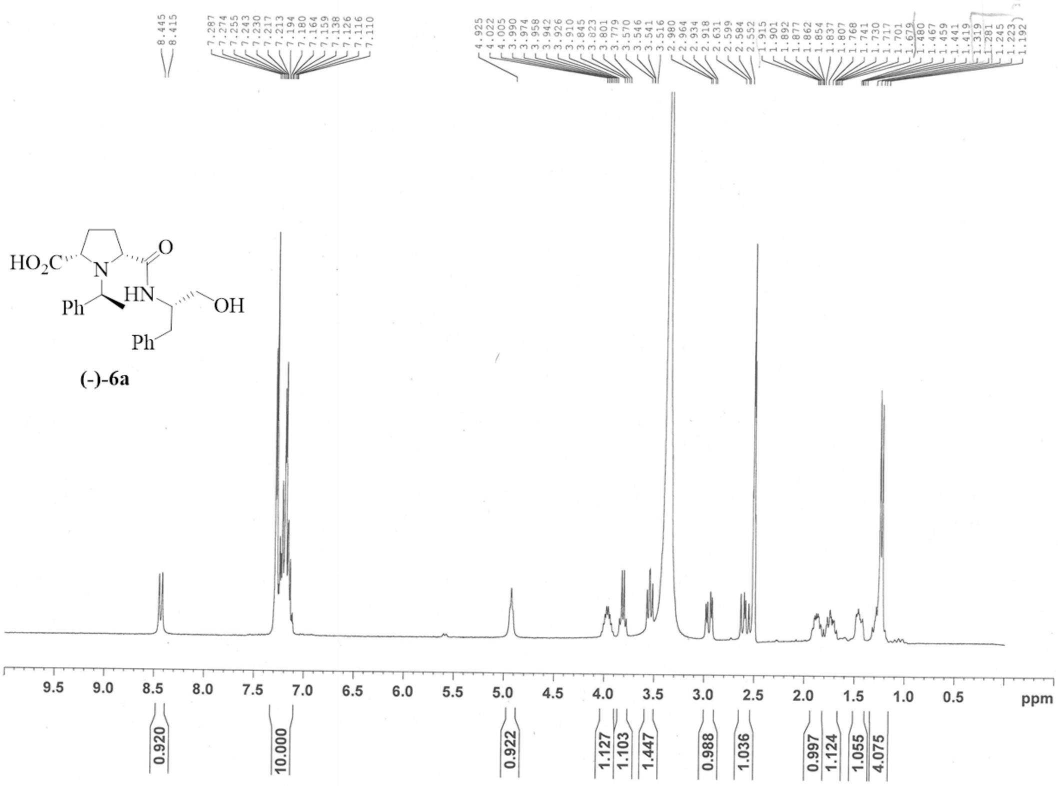

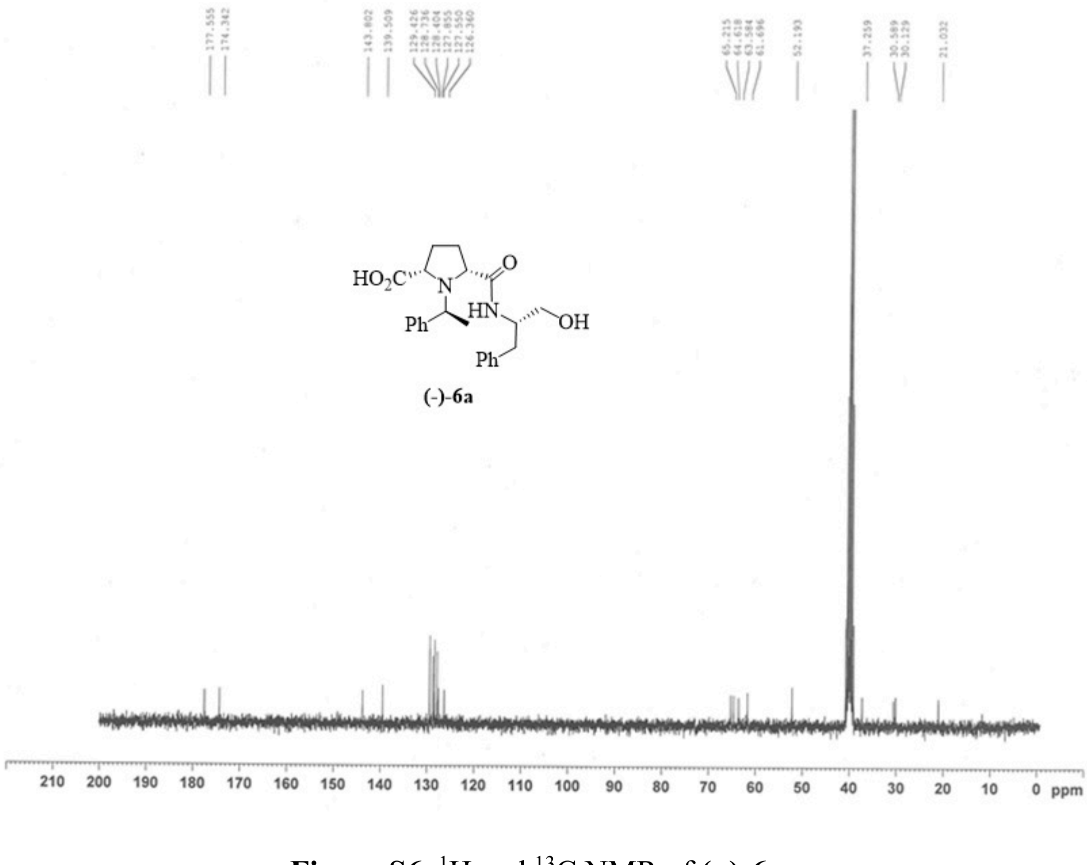

**Figure S6.** $^{1}H$ and $^{13}C$ NMR of **(−)-6a**.

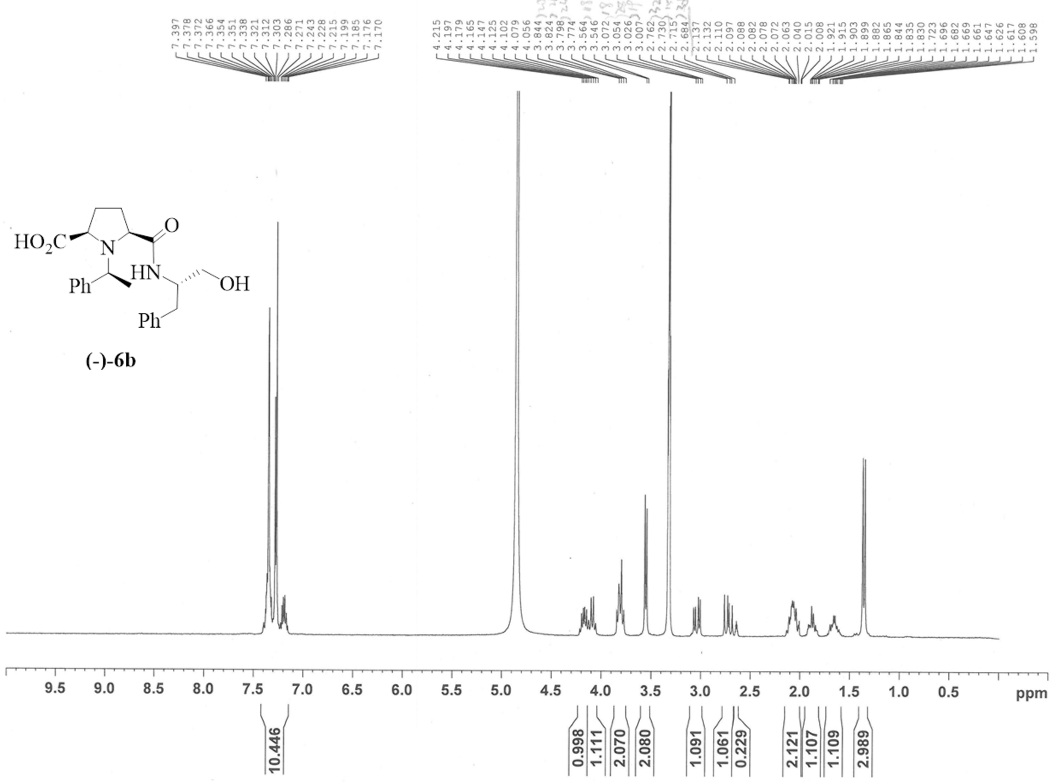

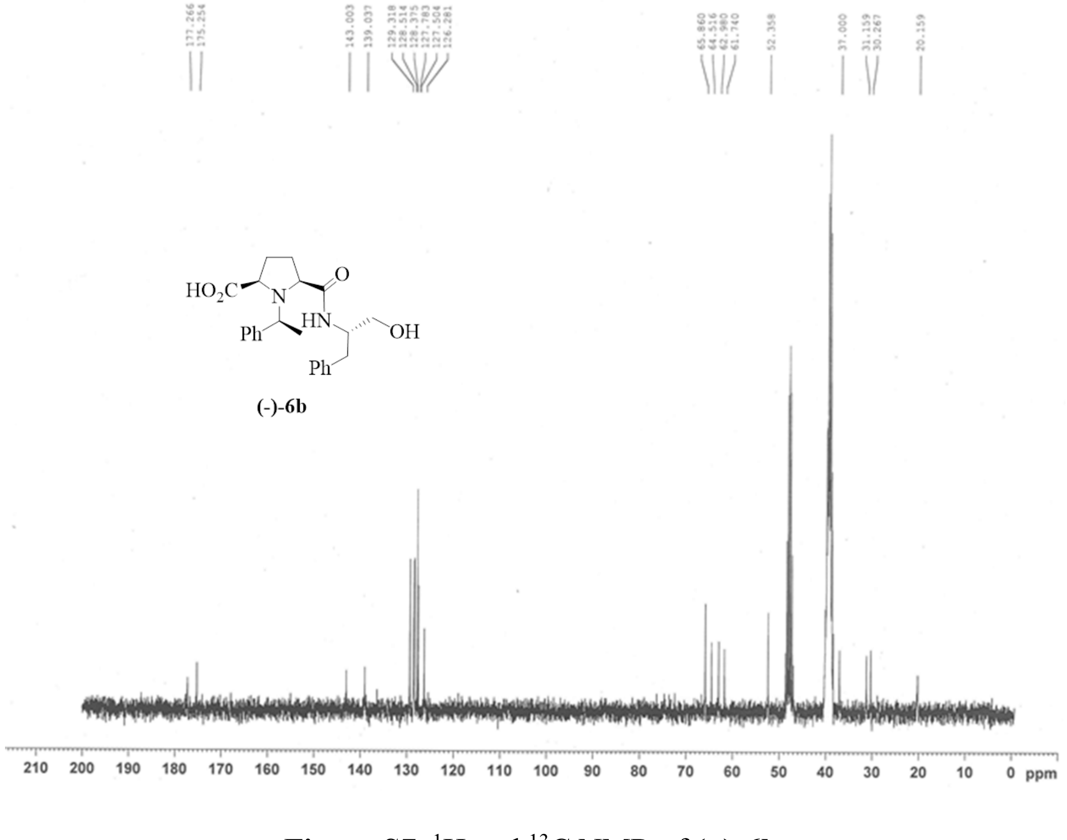

**Figure S7.** $^{1}H$ and $^{13}C$ NMR of **(−)-6b**.

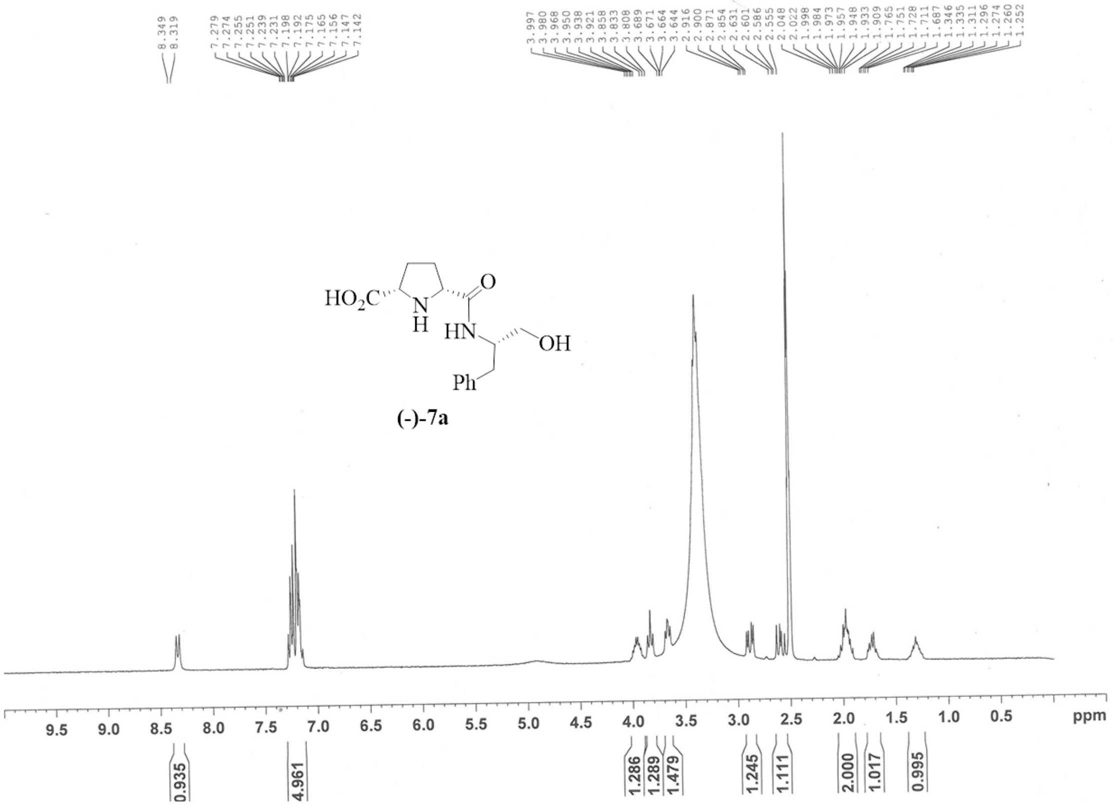

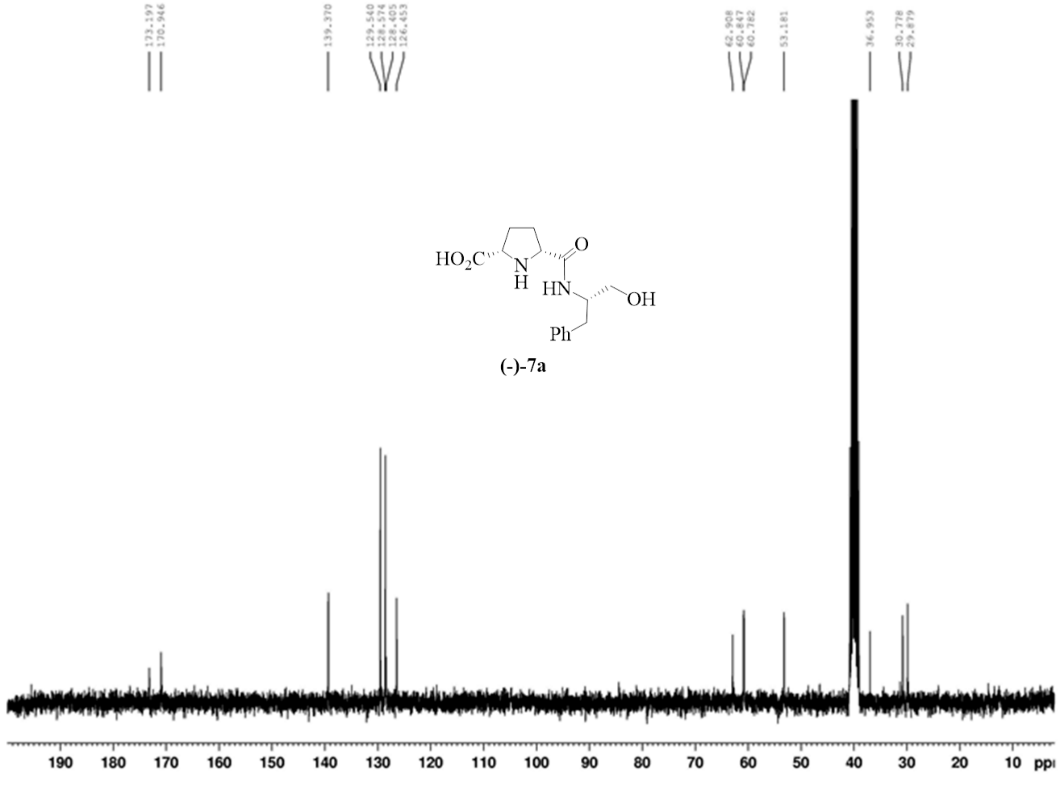

**Figure S8.** $^{1}$H and $^{13}$C NMR of **(−)-7a**.

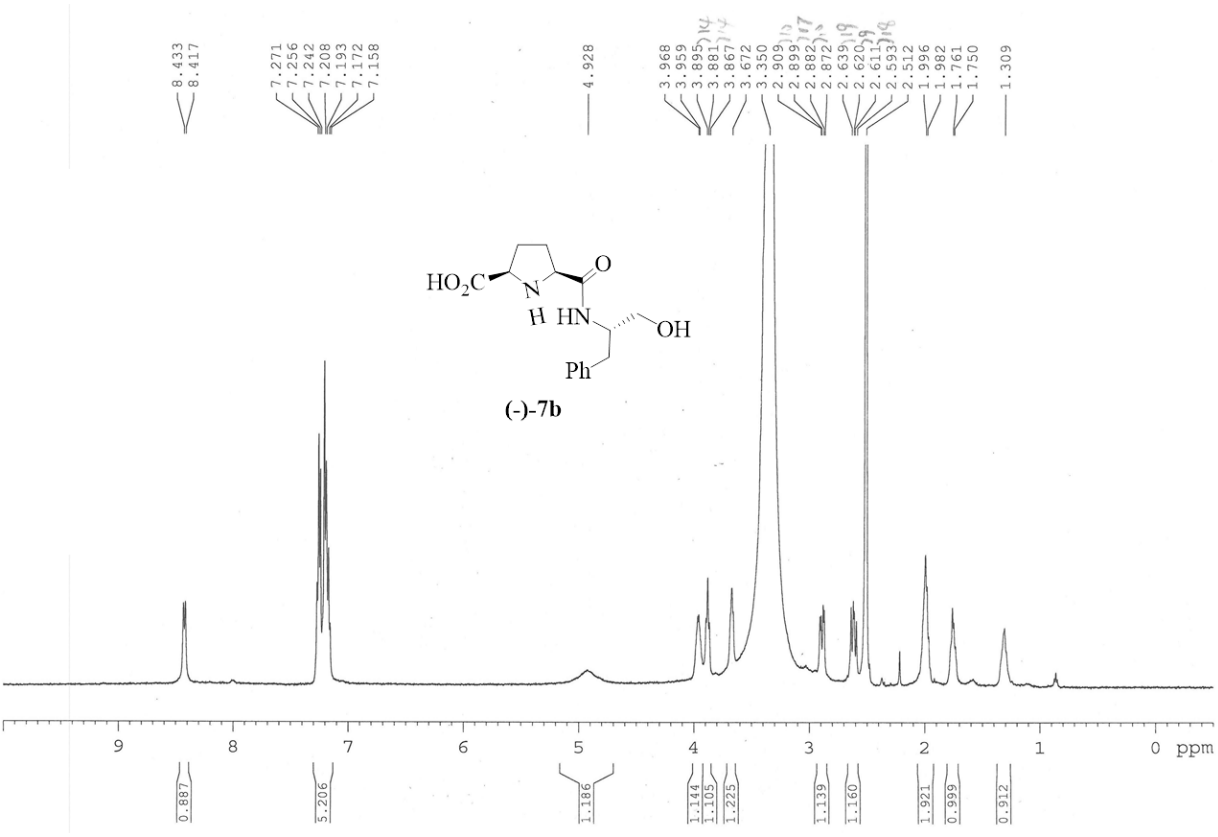

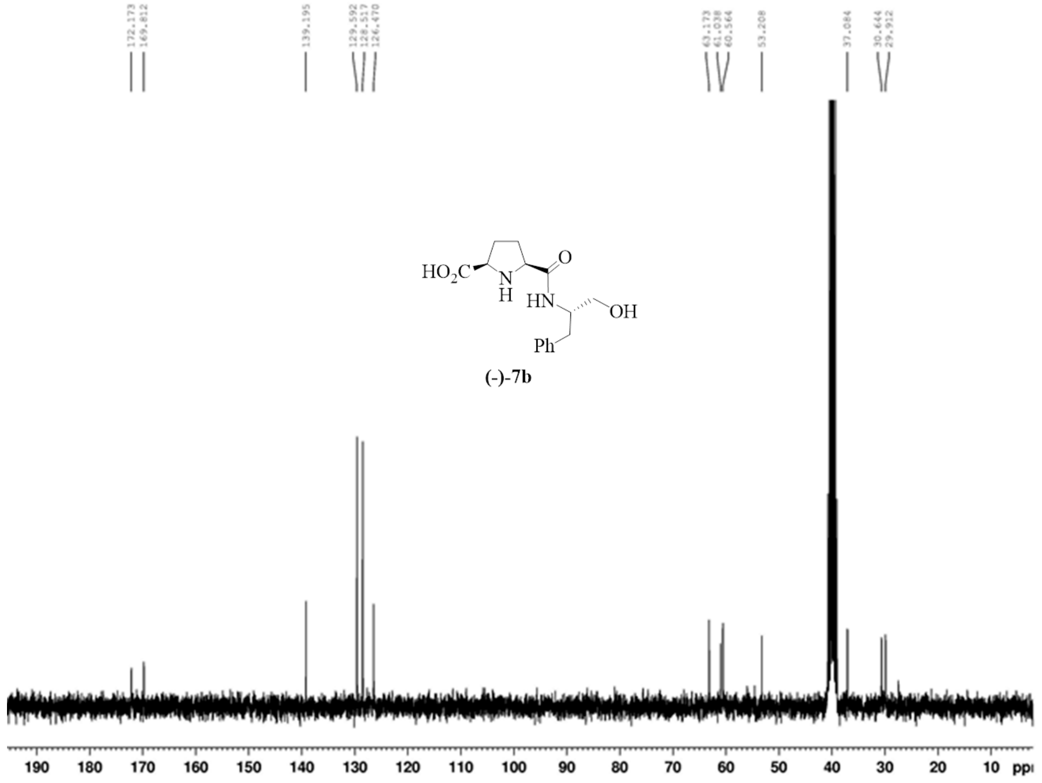

**Figure S9.** $^{1}H$ and $^{13}C$ NMR of **(−)-7b**.
